# Salinity stress accelerates nutrients, dietary fiber, minerals, phytochemicals and antioxidant activity in *Amaranthus tricolor* leaves

**DOI:** 10.1371/journal.pone.0206388

**Published:** 2018-11-01

**Authors:** Umakanta Sarker, Md. Tofazzal Islam, Shinya Oba

**Affiliations:** 1 Laboratory of Field Science, The United Graduate School of Agricultural Science, Faculty of Applied Biological Sciences, Gifu University, Yanagido, Gifu, Japan; 2 Department of Genetics and Plant Breeding, Faculty of Agriculture, Bangabandhu Sheikh Mujibur Rahman Agricultural University, Gazipur, Bangladesh; 3 Department of Biotechnology, Faculty of Agriculture, Bangabandhu Sheikh Mujibur Rahman Agricultural University, Gazipur, Bangladesh; College of Agricultural Sciences, UNITED STATES

## Abstract

Impact of salinity stress were investigated in three selected *Amaranthus tricolor* accessions in terms of nutrients, dietary fiber, minerals, antioxidant phytochemicals and total antioxidant activity in leaves. Salinity stress enhanced biochemical contents and antioxidant activity in *A*. *tricolor* leaves. Protein, ash, energy, dietary fiber, minerals (Ca, Mg, Fe, Mn, Cu, Zn, and Na), β-carotene, ascorbic acid, total polyphenol content (TPC), total flavonoid content (TFC), total antioxidant capacity (TAC) (DPPH and ABTS^+^) in leaves were increased by 18%, 6%, 5%, 16%, 9%, 16%, 11%, 17%, 38%, 20%, 64%, 31%, 22%, 16%, 16%, 25% and 17%, respectively at 50 mM NaCl concentration and 31%, 12%, 6%, 30%, 57%, 35%, 95%, 96%, 82%, 87%, 27%, 63%, 82%, 39%, 30%, 58% and 47%, respectively at 100 mM NaCl concentration compared to control condition. Contents of vitamins, polyphenols and flavonoids showed a good antioxidant activity due to positive and significant interrelationships with total antioxidant capacity. It revealed that *A*. *tricolor* can tolerate a certain level of salinity stress without compromising the nutritional quality of the final product. This report for the first time demonstrated that salinity stress at certain level remarkably enhances nutritional quality of the leafy vegetable *A*. *tricolor*. Taken together, our results suggest that *A*. *tricolor* could be a promising alternative crop for farmers in salinity prone areas- in the tropical and sub-tropical regions with enriched nutritional contents and antioxidant activity.

## Introduction

Salinity is one of the major abiotic stressors which limits crop production and poses a serious threat to global food security. Approximately, 20% percent of the arable land and 50% of total irrigated land have varying levels of salinity [1]. Salinity stress induces a multitude of adverse effects on plants including morphological, physiological, biochemical, and molecular changes. It affects plant growth and development by creating osmotic stress, causing specific ions (Na^+^ and Cl^-^) toxicity, stomatal closure, and reducing the rate of photosynthesis [2].

All these physiological changes in plant under salinity aggravate overproduction of reactive oxygen species (ROS) that interferes normal cellular metabolism and results in oxidative damage by oxidizing proteins, lipids and DNA and other cellular macromolecules [3]. To counterbalance the osmotic stress, plants show variable adaptation processes such as enclosure of stomata, metabolic adjustment, toxic ion homeostasis, and osmotic adjustment [2]. Plants have an excellent network of ROS detoxification system including, either non-enzymatic through protein, carbohydrate, ascorbic acid (AsA), β-carotene and carotenoids, phenolic compounds and flavonoids or through enzymatic antioxidants, such as superoxide dismutase (SOD), peroxidase (GPOX), catalase (CAT), and AsA peroxidase (APX) [3]. Salinity tolerance mechanisms in plants are remarkably varied among the species and even within different accessions of a species.

The leafy vegetables, *A*. *tricolor* comprises an excellent source of proximate and minerals, antioxidant leaf pigments, carotenoids, vitamins, phenolics and flavonoids [4]. Natural antioxidants like leaf pigments, carotenoids, vitamins, phenolics and flavonoids have proven for health benefits as they detoxify ROS (ROS, reactive oxygen species) in the human body [4–5]. Compared to lettuce, *Amaranthus* contains 18 times more vitamin A, 13 times more vitamin C, 20 times more calcium and 7 times more iron. *Amaranthus* leaves contain 3 times more vitamin C, 3 times more calcium and 3 times more niacin than spinach leaves. [6]. It has been rated equal or superior in taste to spinach and is considerably higher in carotenoids (90–200 mg kg^-1^), protein (14–30% on dry weight basis) and ascorbic acid (about 28 mg 100g^-1^) [7]. Minerals are of critical importance in the diet, even though they comprise only 4–6% of the human body. Major minerals are those required in amounts greater than 100 mg per day and they represent 1% or less of body weight. These include calcium, phosphorus, magnesium, sulfur, potassium, chloride, and sodium. Trace minerals are essential in much smaller amounts, less than 100 mg per day, and make up less than 0.01% of body weight. Essential trace elements are zinc, iron, silicon, manganese, copper, fluoride, iodine, and chromium. The major minerals serve as structural components of tissues and function in cellular and basal metabolism and water and acid–base balance [8–9].

Amaranth is a salt tolerant plant [10]. Salinity stress enhances the contents of natural antioxidants in plants [11–13]. Therefore, salt-stressed plants could economically be the potential sources of antioxidants in human lifestyle. The natural antioxidants in diet play an important role in human health as they are involved in defense against several diseases such as cancer, atherosclerosis, arthritis, cataracts, emphysema, retinopathy, neuro-degenerative and cardiovascular diseases [14–17]. *A*. *tricolor* is a well acclimatized leafy popular vegetable to different biotic and abiotic stresses [18]. Various factors such as biological, environmental, biochemical, physiological, ecological and evolutionary processes, and salinity are involved in the quantitative and qualitative improvement of natural antioxidants in this vegetable crop [19]. Salt stress elevated protein, ascorbic acid, phenolics, flavonoids and antioxidant activity and reduced the fat, carbohydrate, sugar, and chlorophyll pigments in *Cichorium spinosum* [11]. Alam et al. [12] observed that in purslane, different doses of salt concentrations increased total polyphenol content (TPC); total flavonoid content (TFC); and FRAP activity by 8–35%, 35%, and 18–35%, respectively. Similarly, in buckwheat sprouts, salinity stress remarkably increased phenolic compounds and carotenoids compared to non-saline condition [13]. *A*. *tricolor* is a popular leafy vegetable in many tropical and subtropical countries. However, no information is available on response of proximate, minerals, vitamins, phenolics, flavonoids and antioxidant activity in the leaves of *A*. *tricolor* accessions to varying leaves of salinity. In a series of earlier studies [20–27], we identified some antioxidant enriched and high yield potential accessions of *A*. *tricolor*. The central hypothesis of this study was that salinity stress may enhance nutritional contents and antioxidant activities in the leaves of *A*. *tricolor*. To test this hypothesis, we investigated the response of proximate, minerals, vitamins, phenolics, flavonoids and antioxidant activity in some selected *A*. *tricolor* accessions to varying levels of salinity stress.

## Materials and methods

### Experimental site, plant materials and experimental conditions

We selected three antioxidants enriched high yield potential accessions (Accession VA3, VA12 and VA14) from 102 accessions of Department of Genetics and Plant Breeding, Bangabandhu Sheikh Mujibur Rahman Agricultural University, based on our earlier studies [20–27]. These accessions were grown in pots of the rain shelter open field of Bangabandhu Sheikh Mujibur Rahman Agricultural University, Bangladesh (AEZ-28, 24^0^23' north latitude, 90^0^08' east longitude, 8.4 m.s.l.). The seeds were sown in plastic pots (15 cm in height and 40 cm length and 30 cm width). The experiment comprised a factorial design of salinity treatment and varieties in a randomized complete block design (RCBD) with three replications. Fertilizer was applied at the rate of 92:48:60 kg ha^−1^ N: P_2_O_5_:K_2_O as a split dose. First, in pot soil, at the rate of 46:48:60 kg ha^−1^ N: P_2_O_5_:K_2_O and second, in 7 days after sowing (DAS) at the rate of 46:0:0 kg ha^−1^ N: P_2_O_5_:K_2_O. Each variety was grouped into three sets and subjected to three salinity stress treatments that are, 100 mM NaCl, 50 mM NaCl, and control or no saline water (NS). Pots were well irrigated by fresh water every day up to 10 DAS of seeds for proper establishment and vigorous growth of seedlings. Imposition of salinity stress treatment was started at 11 DAS and continued up to 40 DAS (edible stage). Saline water (100 mM NaCl and 50 mM NaCl) and fresh water were applied to respective pots once a day. At 40 DAS the leaves of *Amaranthus tricolor* were harvested. All the parameters were measured in six samples.

### Chemicals and reagents used

Solvent: methanol and acetone. Reagents: ascorbic acid, gallic acid, rutin, methanol, DPPH (2, 2-diphenyl1-picrylhydrazyl), ABTS^+^, trolox (6-hydroxy-2, 5, 7, 8-tetramethyl-chroman-2-carboxylic acid), aluminum chloride hexahydrate, sodium carbonate, potassium acetate, Folin-Ciocalteu reagent, H_2_SO_4_, NaOH, HNO_3_, HClO_4_, lanthanum, Caesium chloride, dithiothreitol (DTT) and potassium persulfate. All solvents and reagents used in this study were high purity laboratory products obtained from Kanto Chemical Co. Inc. (Tokyo, Japan) and Merck (Germany).

### Proximate composition in leaf

Moisture content was measured following ASAE standards [28]. Briefly, *A*. *tricolor* leaf samples were oven-dried at 103°C for 72 h, transferred to a desiccator and allowed to cool at room temperature. The sample weights were recorded on a digital balance (Denver Instruments, Denver, Colorado, USA).

Ash, crude fat and crude protein contents were determined by AOAC methods [29]. Ash content was determined by weighing leaf samples before and after heat treatment (550°C for 12 h). Crude fat content was determined according to AOAC method 960.39.

Crude protein was assessed by the micro-Kjeldahl method, with nitrogen to protein conversion factor of 6.25 (AOAC method 976.05). The fiber was determined by ISO method 5498 [30]. First, a sample of leaf powder was boiled in 0.255 M sulfuric acid for 30 min. The resulting insoluble residue was filtered, washed, and boiled in 0.313 M sodium hydroxide. After filtering and washing the sample, it was dried at 130 ± 2°C for 2 h. Weight loss was determined at 350 ± 25°C. The fiber content was expressed relative to the fresh weight (FW). Carbohydrate content (g 100 g^-1^ FW) was calculated by subtracting the sum of percent moisture, ash, crude fat and crude protein from 100. Gross energy was determined using a bomb calorimeter according to ISO method 9831.

### Estimation of leaf mineral contents

Leaves of *A*. *tricolor* were dried at 70°C in a well-ventilated drying oven for 24 hours. Dried leaf of *A*. *tricolor* ground finely in a mill and passed through an 841 microns’ screen, then portions of the dried tissues were analyzed for the following macronutrients (Ca, Mg and K) and microelements (Fe, Mn, Cu, Zn and Na). All macronutrients and microelements were extracted after the dissolution of the *A*. *tricolor* samples by nitric-perchloric acid digestion [31]. Nitric-perchloric acid digestion was performed by adding 0.5 g of the dried samples with 400 ml of nitric acid (65%), 40 ml of perchloric acid (70%) and 10 ml of sulphuric acid (96%) in the presence of carborundum beads. After nitric-perchloric acid digestion, the solution was appropriately diluted and P analysis was performed in triplicate according to the Ascorbic Acid Method. In acidic medium, orthophosphates formed a yellow-colored complex with molybdate ions and, after addition of ascorbic acid and Sb, a blue-colored phosphomolybdenum complex was formed. Absorbance was taken according to the method described by Temminghoff and Houba [32] at wavelength of 766.5 nm (K), 422.7 nm (Ca), 285.2 nm (Mg), 248.3 nm (Fe), 279.5 nm (Mn), 324.8 nm (Cu), 213.9 nm (Zn) and 589.0 nm (Na) by atomic absorption spectrophotometry (AAS) (Hitachi, Tokyo, Japan). For calibration, AAS standard solutions (1,000 mg l^-1^ in 5% HNO_3_) were purchased from Merck, Germany. Finally, interferences were controlled by the addition of lanthanum and caesium chloride (0.1%) to samples and standards.

### Determination of β-carotene

The extraction and estimation of β-carotene were performed according to the protocol described by Sarker and Oba [33]. During the extraction process, 500 mg of fresh leaf sample was ground in 10 ml of 80% acetone and centrifuged at 10,000 rpm for 3–4 min. The supernatant was removed and brought up to 20 ml in a volumetric flask, and the absorbance was measured at 510 nm and 480 nm spectrophotometrically using a Hitachi U-1800 instrument (Hitachi, Tokyo, Japan). Data were expressed as mg β-carotene per kg fresh weight.

The β-carotene content was calculated using the following formula:

Amountofβ‑carotene=7.6(Abs.at480)‑1.49(Abs.at510)×Finalvolume/(1000×freshweightofleaftaken)

### Ascorbic acid assay

The total ascorbic acid defined as ascorbic acid (AsA) and dehydroascorbate (DHA) acid was assessed by spectrophotometric detection on fresh plant tissues. The assay is based on the reduction of Fe_3_^+^ to Fe_2_^+^ by AsA and the spectrophotometric (Hitachi, U-1800, Tokyo, Japan) detection of Fe_2_^+^ complexes with 2, 2-dipyridyl [33]. DHA is reduced to AsA by pre-incubation of the sample with dithiothreitol (DTT). The absorbance of the solution was measured at 525 nm spectrophotometrically using a Hitachi U1800 instrument (Hitachi, Tokyo, Japan). Data were expressed as mg ascorbic acid per kg fresh weight.

### Extraction of samples for TPC, TFC and TAC analysis

Amaranth leaves were harvested at the edible stage (35 Days after sowing) and air dried (in shade) for chemical analysis. One gram of dried leaves from each cultivar was ground and suspended in 40 ml of 90% aqueous methanol in a tightly capped bottle (100 ml), which was then placed in a shaking water bath (Thomastant T-N22S, Thomas Kagaku Co. Ltd., Japan) for 1 h. Then, the extract was filtered through further analytical assays of total polyphenol content, total antioxidant activity, total flavonoids content.

### Determination of total polyphenols (TPC)

The total phenolic content of *A*. *tricolor* was determined using the Folin-Ciocalteu reagent method described by Sarker and Oba [34] with gallic acid as a standard phenolic compound. Briefly, 50 μl of the leaf extract solution was placed in a test tube along with 1 ml of the Folin-Ciocalteu reagent (previously diluted 1:4, reagent: distilled water) and then mixed thoroughly. After 3 min, 1 ml of Na_2_CO_3_ (10%) was added, and the mixture allowed to stand for 1 h in the dark. The absorbance was measured at 760 nm spectrophotometrically using a Hitachi U1800 instrument (Hitachi, Tokyo, Japan). The concentration of total phenolic compounds in the leaf extracts was determined using an equation obtained from a standard gallic acid graph. The results are expressed as mg gallic acid equivalent (GAE) kg^-1^ dw.

### Determination of total flavonoid content (TFC)

The total flavonoid content of *A*. *tricolor* extract was determined using the aluminum chloride colorimetric method described by Sarker and Oba [34]. For this assay, 500 μl of leaf extract was transferred to a test tube along with 1.5 ml of methanol, 0.1 ml of 10% aluminum chloride, 0.1 ml of 1 M potassium acetate and 2.8 ml of distilled water. After 30 min at room temperature, the absorbance of the reaction mixture was measured at 415 nm spectrophotometrically using a Hitachi U1800 instrument (Hitachi, Tokyo, Japan). Rutin was used as the standard compound, and TFC is expressed as mg rutin equivalent (RE) kg^-1^ dw.

### Measurement of total antioxidant capacity (TAC)

Antioxidant activity was measured using the diphenyl-picrylhydrazyl (DPPH) radical degradation method [35]. Briefly, 10 μl of leaf extract solution (in triplicate) was placed in test tubes along with 4 ml of distilled water and 1 ml of 250 μM DPPH solution. The tubes were mixed and allowed to stand for 30 min in the dark before the absorbance was read at 517 nm spectrophotometrically using a Hitachi U1800 instrument (Hitachi, Tokyo, Japan). For the ABTS^+^ assay the method described by Sarker and Oba [35] was followed. The stock solutions included 7.4 mM ABTS^+^ solution and 2.6 mM potassium persulfate solution. The working solution was prepared by mixing the two stock solutions in equal quantities and allowing them to react for 12 h at room temperature in the dark. A 150 μl sample of leaf extract was allowed to react with 2850 μl of ABTS^+^ solution (1 ml ABTS^+^ solution mixed with 60 ml methanol) for 2 h in the dark. The absorbance was taken at 734 nm spectrophotometrically against methanol using a Hitachi U1800 instrument (Hitachi, Tokyo, Japan). Antioxidant activity was calculated as the percent of inhibition of DPPH and ABTS^+^ relative to the control using the following equation:

Antioxidantactivity(%)=(Abs.blank‑Abs.sample/Abs.blank)×100

Where, Abs. blank is the absorbance of the control reaction (10 μl methanol for TAC (DPPH), 150 μl methanol for TAC (ABTS^+^) instead of leaf extract) and Abs. sample is the absorbance of the test compound. Trolox was used as the reference standard, and the results were expressed as mg trolox equivalent kg^-1^ dw.

### Statistical analysis

The results were reported as the mean ± SD of three separate replications (six separate measurements of each replication). The data were also statistically analyzed by ANOVA using Statistix 8 software, and the means were compared by Duncan’s multiple range (DMRT) test for 0.1% level of probability.

## Results

### Effect of salinity on proximate composition in *A*. *tricolor* leaves

**Fig 1:**
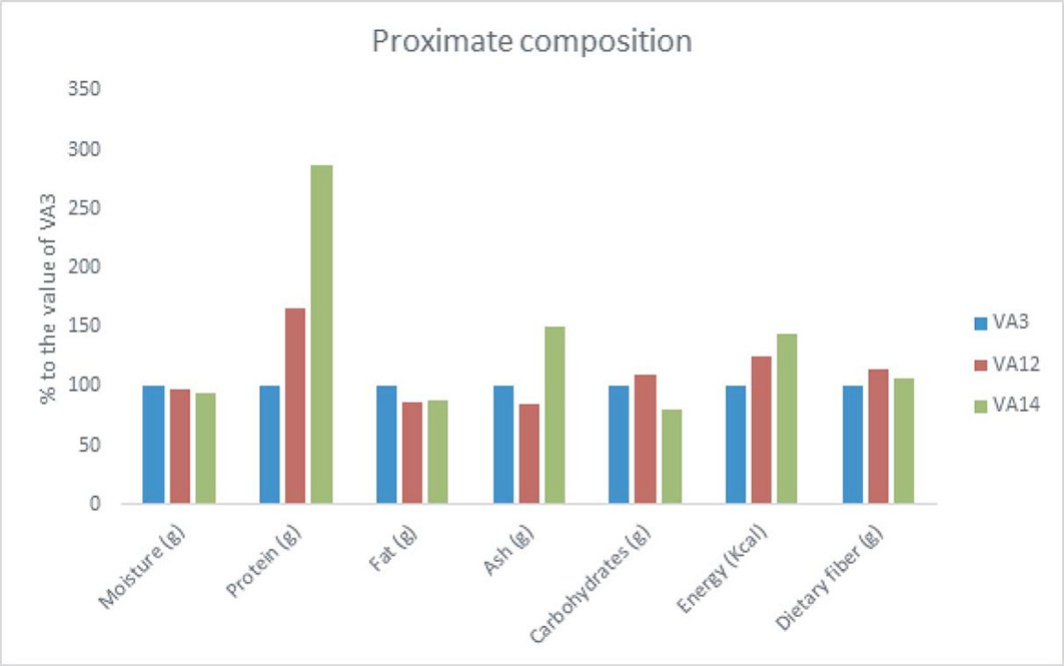
Influence of salinity on proximate composition (g 100 g^-1^) (% to the value of VA3) in three selected *A*. *tricolor* accessions.

The proximate compositions of *A*. *tricolor* leaves were significantly varied by accessions, salinity levels and accession × salinity stress interactions (Table 1). Among the tested accessions, VA14 had the highest protein (7.25 g 100 g^-1^), ash content (5.78 g 100 g^-1^) energy (54.52 Kcal 100 g^-1^) and the lowest moisture content (81.56 g 100 g^-1^). However, accession VA12 gave the highest contents of dietary fiber (8.28 g 100 g^-1^) and carbohydrates (7.06 g 100 g^-1^). The highest fat content (0.36 g 100 g^-1^) was recorded in accession VA3. The accession, VA14 had 187%, 50%, and 44% higher protein, ash, and energy contents, respectively compared to the accession VA3. Accession VA12 had 10% and 25% higher carbohydrates and energy, respectively than accession VA3. (Fig 1).

**Table 1:** Salinity effect on proximate composition (per 100 g fresh weight) in three selected *A*. *tricolor* accessions.

Treatment	Moisture (g)	Protein (g)	Fat (g)	Ash (g)	Carbohydrates (g)	Energy (Kcal)	Dietary fiber (g)
**Accession × Salinity stress (SS)**							
VA3 × NS	88.16 ± 2.13a	2.15 ± 0.04i	0.43 ± 0.01a	3.53 ± 0.05f	5.73 ± 0.02e	33.60 ± 1.12i	6.45 ± 0.07h
VA3 × MSS	87.23 ± 2.08b	2.27 ± 0.04h	0.38 ± 0.02b	3.86 ± 0.06e	6.27 ± 0.05d	35.59 ± 1.20h	7.22 ± 0.09f
VA3 × SSS	85.29 ± 1.56d	3.15 ± 0.02g	0.27 ± 0.03g	4.11 ± 0.05d	7.17 ± 0.04a	41.77 ± 0.89g	8.11 ± 0.08d
VA12 × NS	86.22 ± 2.07c	3.74 ± 0.03f	0.35 ± 0.01c	2.68 ± 0.06h	7.01 ± 0.07c	44.57 ± 1.24f	7.22 ± 0.06f
VA12 × MSS	85.12 ± 1.67d	4.25 ± 0.04e	0.31 ± 0.02e	3.24 ± 0.07g	7.07 ± 0.06b	46.72 ± 1.32e	8.37 ± 0.08c
VA12 × SSS	84.23 ± 1.59e	4.55 ± 0.07d	0.27 ± 0.04g	3.86 ± 0.04e	7.09 ± 0.07b	47.72 ±1.46d	9.24 ± 0.05a
VA14 × NS	82.17 ± 1.54f	6.25 ± 0.06c	0.35 ± 0.02c	5.46 ± 0.05c	5.78 ± 0.07e	51.51 ± 0.89c	6.76 ± 0.04g
VA14 × MSS	81.44 ± 2.16g	7.36 ± 0.05b	0.32 ± 0.01d	5.76 ± 0.06b	5.11 ± 0.06f	53.92 ± 0.82b	7.83 ± 0.08e
VA14 × SSS	81.07 ± 1.57g	8.16 ± 0.03a	0.28 ± 0.03f	6.12 ± 0.08a	4.38 ± 0.05i	54.52 ± 1.26a	8.75 ± 0.08b
**Accession**							
VA3	86.89 ± 1.65a	2.52 ± 0.02c	0.36 ± 0.02a	3.83 ± 0.05b	6.39 ± 0.04b	36.99 ± 0.99c	7.26 ± 0.05c
VA12	85.19 ± 1.86b	4.18 ± 0.04b	0.31 ± 0.01c	3.26 ± 0.08c	7.06 ± 0.03a	46.34 ± 1.14b	8.28 ± 0.07a
VA14	81.56 ±1.92c	7.25 ± 0.06a	0.32 ± 0.03b	5.78 ± 0.07a	5.09 ± 0.06c	53.32 ± 1.13a	7.78 ± 0.09b
**Salinity stress (SS)**							
NS	85.52 ± 1.58a	4.05 ± 0.03c	0.38 ± 0.04a	3.89 ± 0.06c	6.17 ± 0.07b	43.23 ± 1.08c	6.81 ± 0.05c
MSS	84.60 ± 1.49b	4.63 ± 0.05b	0.34 ± 0.02b	4.29 ± 0.08b	6.15 ± 0.06b	45.41 ± 1.15b	7.81 ± 0.09b
SSS	83.53 ± 1.74c	5.29 ± 0.06a	0.27 ± 0.03c	4.70 ± 0.07a	6.21 ± 0.08a	48.00 ±1.18a	8.70 ± 0.07a
**Significance**							
Accession	***	***	***	***	***	***	***
SS	***	***	***	***	***	***	***
Accession × SS	***	***	***	***	***	***	***

SS, Salinity stress; NS, No saline water; MSS, Moderate salinity stress, SSS, Severe salinity stress, Values are means of six replicates and different letters are differed significantly by Duncan Multiple Range Test (P < 0.001).

**Fig 2:**
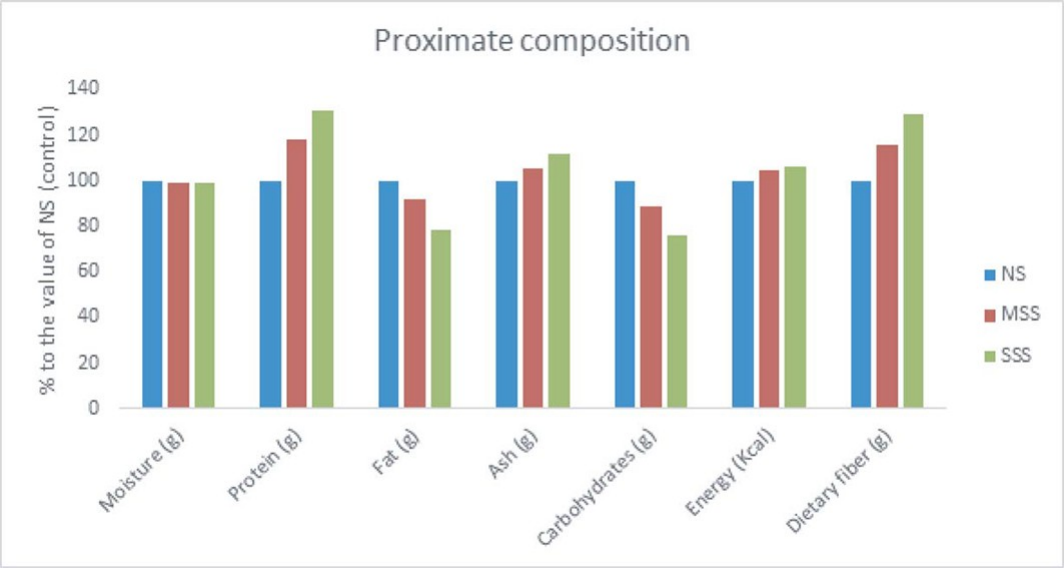
Changes of proximate composition (g 100 g^-1^) (% to the value of NS or control) in the leaves of *A*. *tricolor* accessions under three salinity stress levels. NS or control, no saline water; MSS, moderate salinity stress; and SSS, severe salinity stress.

The contents of protein, ash, energy and dietary fiber in *A*. *tricolor* leaves increased by salinity stress in a level-dependent manner (Fig 2). The increment of protein, ash, energy and dietary fiber contents in *A*. *tricolor* by moderate salinity stress (MSS) and severe salinity stress (SSS) were 17, 5, 4 and 15% and 30, 12, 5 and 29%, respectively over no salinity (NS) or control condition. Among salinity stress, NS or control treatment exhibited the highest moisture and fat content, however, moisture and fat contents were the lowest at the SSS conditions. A significant reduction in moisture and fat contents was observed with the increment of salinity stress (control or NS > MSS > SSS). Contents of protein, ash, energy and dietary fiber in plants at SSS conditions were the highest among the salinity stress treatment. The lowest values of these plant parameters were recorded in the control or NS.

The highest carbohydrates content (6.21 g 100 g^-1^) was found in plants grown under SSS, whereas the lowest values (6.15 and 6.17 g 100 g^-1^) of this parameter were found in NS and MSS treatments, respectively.

In the case of accession × salinity stress interaction, accession VA3 had the highest moisture content (86.16 g 100 g^-1^) at no salinity stress condition. Both MSS and SSS reduced the moisture content at the lowest levels (81.07 and 81.44 g 100 g^-1^) in accession VA14 that were followed by followed by accessions VA14 and VA14 under NS and SSS conditions, respectively. The highest protein content (8.16 g 100 g^-1^) was recorded in accession VA14 under SSS followed by VA14 (7.36 g 100 g^-1^) and VA14 (6.25 g 100 g^-1^) under MSS and NS conditions, respectively. The lowest protein content (2.15 g 100 g^-1^) was found in accession VA3 under nonsaline treatment, which was almost similar to VA3 under MSS (2.27 g 100 g^-1^). The fat contents in *A*. *tricolor* varied from 0.43 to 0.27 g 100 g^-1^. The highest fat content (0.43 g 100 g^-1^) was recorded in accession VA3 when no salinity stress was given to the plants whereas fat content in VA3 and VA12 was as low as 0.27 g 100 g^-1^ under SSS conditions. Accession VA3 also had the highest carbohydrate content (7.17 g 100 g^-1^) when plants were treated with SSS. On the other hand, VA14 under MSS (5.11 g 100 g^-1^) had the lowest carbohydrates content.

The ash content in *A*. *tricolor* accessions varied (2.68 to 6.12 g 100 g^-1^) under varying levels of salt stress. The highest ash content (6.12 g 100 g^-1^) was recorded in accession VA14 at SSS conditions. The lowest as content (2.68 g 100 g^-1^) was found in accession VA12 under non-saline control. The energy contents in *A*. *tricolor* plants ranged from 33.60 to 54.52 Kcal 100 g^-1^. The accession VA14 exhibited the highest energy (54.52 Kcal 100 g^-1^) under SSS followed by VA14 under MSS and NS or control, respectively. In contrast, the lowest energy was recorded in VA3 under NS or control. The energy was significantly increased to the increment of salinity stress in the following order: NS or control < MSS < SSS. The accession VA12 under SSS had the highest fiber content (9.24 g 100 g^-1^) followed by VA14 under SSS (8.75 g 100 g^-1^), VA12 under MSS (8.37 g 100 g^-1^), VA3 under SSS (8.11 g 100 g^-1^). Alternatively, VA3 under NS or control had the lowest fiber content (6.45 g 100 g^-1^).

### Effects of salinity on mineral (macro and micro elements) composition in leaves

The mineral compositions of *A*. *tricolor* accessions significantly varied with varying levels of salinity stress and accession × salinity stress interactions (Table 2).

**Table 2:** Salinity stress on mineral composition (macro mg g^-1^ FW and micro μg g^-1^ FW nutrient elements) in the leaves of three selected *A*. *tricolor* accessions.

	Macroelements (mg g^-1^ FW)			Microelements (μg g^-1^ FW)				
Treatment	Ca	Mg	K	Fe	Mn	Cu	Zn	Na
**Accession × Salinity stress (SS)**								
VA3 × NS	2.05 ± 0.07h	2.55 ± 0.02e	4.58 ± 0.02d	10.26 ± 0.08i	10.23 ± 0.06i	0.98 ± 0.02i	10.58 ± 0.08i	62.55 ± 0.14i
VA3 × MSS	3.16 ± 0.05f	3.42 ± 0.03c	3.44 ± 0.01g	12.25 ± 0.09h	16.47 ± 0.04f	1.22 ± 0.01g	12.45 ± 0.07h	126.45 ± 0.21f
VA3 × SSS	4.26 ± 0.04c	4.72 ± 0.04a	2.25 ± 0.03i	14.62 ± 0.08f	21.31 ± 0.04b	1.76 ± 0.03e	16.35 ± 0.05d	246.55 ± 0.25b
VA12 × NS	2.37 ± 0.03g	2.50 ± 0.01e	4.33 ± 0.04e	13.35 ± 0.07g	11.22 ± 0.05h	1.12 ± 0.02h	13.13 ± 0.06g	74.63 ± 0.23h
VA12 × MSS	3.57 ± 0.07d	3.42 ± 0.05c	3.76 ± 0.03f	17.56 ± 0.05 d	13.55 ± 0.08g	1.58 ± 0.01f	15.63 ± 0.08e	148.94 ± 0.25d
VA12 × SSS	4.85 ± 0.06b	3.91 ± 0.06b	2.36 ± 0.02h	22.78 ± 0.09b	17.62 ± 0.06d	2.15 ± 0.02d	19.34 ± 0.09b	320.66 ± 0.26a
VA14 × NS	3.34 ± 0.05e	2.47 ± 0.02e	7.58 ± 0.04a	16.67 ± 0.08e	16.66 ± 0.07e	2.35 ± 0.03c	14.76 ± 0.08f	80.63 ± 0.28g
VA14 × MSS	3.62 ± 0.05d	2.86 ± 0.04d	6.11 ± 0.01b	18.53 ± 0.05c	19.43 ± 0.03c	3.25 ± 0.04b	17.65 ± 0.07c	132.45 ± 0.27e
VA14 × SSS	5.24 ± 0.06a	3.35 ± 0.03c	5.67 ± 0.03c	32.46 ± 0.07a	32.58 ± 0.05a	4.28 ± 0.02a	27.56 ± 0.09a	182.95 ± 0.29c
**Accession**								
VA3	3.16 ± 0.07c	3.57 ± 0.05a	3.42 ± 0.02c	12.38 ± 0.06c	16.00 ± 0.05b	1.32 ± 0.02c	13.13 ± 0.06c	145.18 ± 0.24b
VA12	3.60 ± 0.02b	3.28 ± 0.06b	3.49 ± 0.03b	17.90 ± 0.08b	14.13 ± 0.06c	1.62 ± 0.03b	16.03 ±0.08b	181.41 ± 0.28a
VA14	4.07 ± 0.03a	2.90 ± 0.01c	6.45 ± 0.02a	22.55 ± 0.09a	22.89 ± 0.05a	3.30 ± 0.01 a	19.99 ±0.08a	132.01 ± 0.27c
**Salinity stress (SS)**								
NS	2.58 ± 0.05c	2.51 ± 0.02c	5.50 ± 0.01a	13.43 ± 0.08c	12.70 ± 0.04c	1.49 ± 0.02c	12.82 ± 0.07c	72.60 ± 0.24c
MSS	3.45 ± 0.04b	3.24 ± 0.04b	4.44 ± 0.02b	16.11 ± 0.07b	16.48 ± 0.03b	2.02 ± 0.01b	15.25 ± 0.09b	135.94 ± 0.26b
SSS	4.78 ± 0.03a	3.99 ± 0.06a	3.43 ± 0.04c	23.29 ± 0.05a	23.84 ± 0.05a	2.73 ± 0.03a	21.08 ± 0.08a	250.06 ± 0.28a
**Significance**								
Accession	***	***	***	***	***	***	***	***
SS	***	***	***	***	***	***	***	***
Accession × SS	***	***	***	***	***	***	***	***

SS, Salinity stress; NS, No saline water; MSS, Moderate salinity stress, SSS, Severe salinity stress, Values are means of six replicates and different letters are differed significantly by Duncan Multiple Range Test (***, P < 0.001).

**Fig 3:**
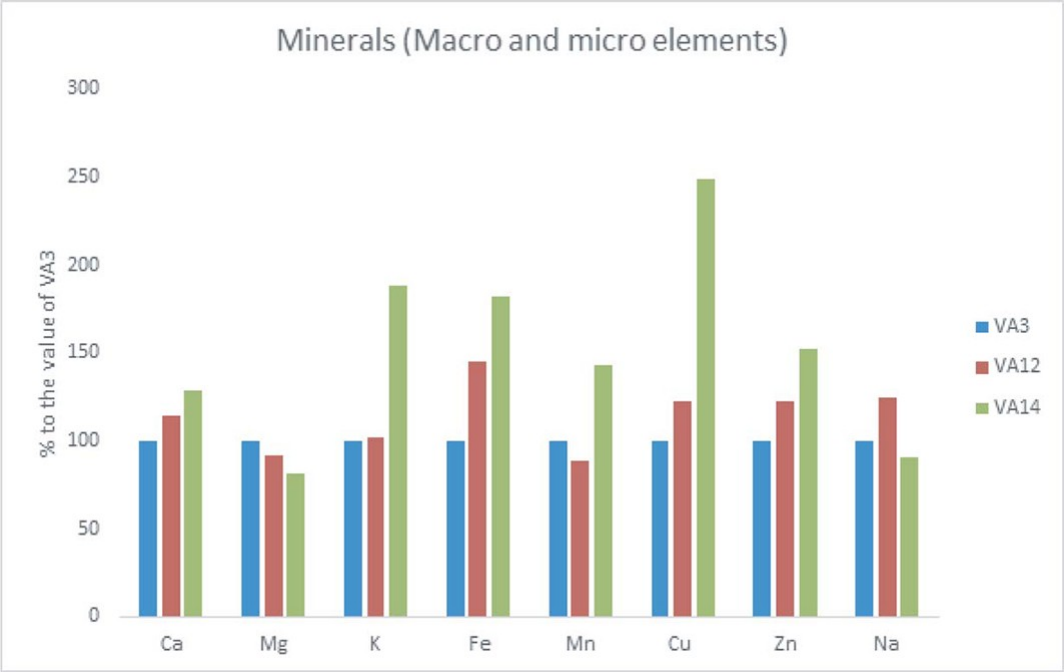
Minerals (macro mg g^-1^ and micro μg g^-1^ nutrient elements) contents (% to the value of VA3) in the leaves of three selected *A*. *tricolor* accessions.

Among the tested accessions, the highest Ca, K, Fe, Mn, Cu and Zn contents were found in VA14. However, VA3 had the highest Mg content whereas the highest content of Na was recorded in VA12. In contrast, VA3 exhibited the lowest contents of Ca, K, Fe, Cu and Zn. Similarly, VA14 had the lowest Mg and Na content and VA12 showed the lowest Mn content. Accession VA14 exhibited 28%, 88%, 82%, 43%, 49% and 52% higher Ca, K, Fe, Mn, Cu and Zn content, respectively compared to VA3. Accession VA12 had 24% more Na content compared to VA3. (Fig 3).

**Fig 4:**
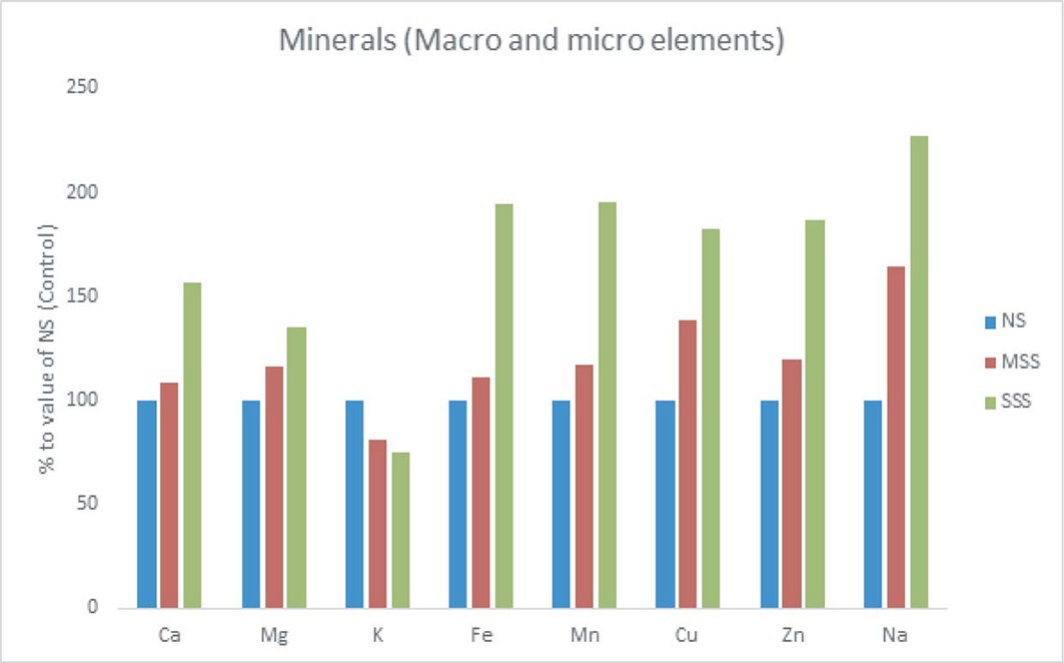
Comparison of minerals (macro mg g^-1^ and micro μg g^-1^ nutrient elements) (% to the value of NS or control) contents in *A*. *tricolor* leaves under three salinity levels. NS or control, no salinity stress; MSS, moderate salinity stress; and SSS, severe salinity stress.

Across the salinity stresses, Ca, Mg, Fe, Mn, Cu, Zn and Na contents in leaves were sharply and significantly increased with the increment of salinity stress in the following order: NS or control < MSS < SSS. At MSS and SSS, the rate of the increment of Ca, Mg, Fe, Mn, Cu, Zn and Na were (8%, %, 11%, 16%, 38%, 19%, 64%) and (57%, 35%, 95%, 96%, 82%, 87%, 27%), respectively, over NS or control (Fig 4). Further, it was noted that the severity of salinity stress leads to a significant reduction in K content in the following order: NS or control > MSS > SSS. In MSS and SSS, K reduced 19% and 25%, respectively over NS or control. (Fig 4). SSS had the highest Ca, Mg, Fe, Mn, Cu, Zn and Na content while, the lowest Ca, Mg, Fe, Mn, Cu, Zn and Na content were demonstrated in NS or control. On the contrary, the highest K content was documented in NS or control and the lowest K content was observed in SSS.

Considering the accession × salinity stress interaction, the highest Ca content was noted in VA14 under SSS (5.24 mg g^-1^ FW) followed by VA12 under SSS and VA3 under SSS. In contrast, VA3 under NS or control (2.05 mg g^-1^ FW) displayed the lowest Ca content. Mg content ranged from 2.47 to 4.72 mg g^-1^ FW. The highest Mg content was observed in VA3 under SSS (4.72 mg g^-1^ FW) followed by VA12 under SSS, VA12 under MSS and VA3 under MSS. In contrast, VA14, VA12 and VA3 under NS or control (2.47, 2.50 and 2.55 mg g^-1^ FW) displayed the lowest Mg content. The range of K content was 2.25 to 7.58 mg g^-1^ FW. VA14 under NS had the highest K content (7.58 mg g^-1^ FW) followed by VA14 under SSS and VA14 under MSS, while the lowest K content was noticed in VA3 under NS (2.55 mg g^-1^ FW). The Fe content ranged from 10.26 to 32.46 μg g^-1^ FW. The highest Fe content was observed in VA14 under SSS (32.46 μg g^-1^ FW), whereas, VA3 under NS (10.26 μg g^-1^ FW) exhibited the lowest Fe content. Mn content ranged from 10.23 to 32.58 μg g^-1^ FW. Accession VA14 under SSS exhibited the highest Mn content (32.58 μg g^-1^ FW), while, VA3 under NS had the lowest Mn content (10.23 μg g^-1^ FW). Accession VA14 under SSS had the highest Cu content (4.28 μg g^-1^ FW). In contrast, the lowest Cu content (0.98 μg g^-1^ FW) was recorded in VA3 under NS. Zn content ranged from 10.58 to 27.56 μg g^-1^ FW. Accession VA14 under SSS showed the highest Zn content (27.56 μg g^-1^ FW), whereas, the lowest Zn content (10.58 μg g^-1^ FW) was reported on VA3 under NS. The highest Na content was detected in VA12 under SSS (320.66 μg g^-1^ FW) which ranged from 62.55 to 320.66 μg g^-1^ FW. The lowest Na content (62.55 μg g^-1^ FW) was recorded in VA3 under NS.

### Salinity stress enhances β-carotene, ascorbic acid, TPC, TFC and TAC in *A*. *tricolor* leaves

Total polyphenol content (TPC), β-carotene, total flavonoid content (TFC), ascorbic acid, and total antioxidant capacity (TAC) in *A*. *tricolor* leaves were significantly affected by accession, salt concentration and accession × salt concentration interactions (Table 3).

**Table 3:** Salinity effects on antioxidant phytochemicals and antioxidant capacity in three selected *A*. *tricolor* accessions.

Treatment	β-carotene (mg kg^-1^)	Ascorbic acid (mg kg^-1^)	Total polyphenol content (GAE mg kg^-1^ dw)	Total flavonoid content (RE mg kg^-1^ dw)	Total antioxidant capacity (DPPH) (TEAC mg kg^-1^ dw)	Total antioxidant capacity ABTS+) (TEAC mg kg^-1^ dw)
**Accession × Salinity stress (SS)**						
VA3 × NS	8.28 ± 0.09g	165.74 ± 2.15i	28.25 ± 0.24e	110.45 ± 1.47i	32.56 ± 0.15d	55.56 ± 0.37g
VA3 × MSS	8.74 ± 0.11e	212.86 ± 2.47h	30.63 ± 0.31c	129.85 ±1.52h	33.45 ± 0.24c	58.75 ± 0.45e
VA3 × SSS	10.26 ± 0.12c	286.63 ± 3.12g	32.45 ± 0.42b	154.17 ± 2.02g	35.42 ± 0.16b	63.52 ± 0.57c
VA12 × NS	7.78 ± 0.15h	984.65 ± 3.51f	20.26 ± 0.33i	340.65 ± 2.48d	23.52 ± 0.27h	52.35 ± 0.62h
VA12 × MSS	8.44 ± 0.07f	1052.74 ± 3.24e	22.49 ± 0.38h	385.96 ± 3.07b	24.55 ± 0.23g	56.38 ± 0.53f
VA12 × SSS	10.32 ± 0.14c	1225.53 ± 2.87d	26.36 ± 0.54f	422.54 ± 1.95a	26.88 ± 0.24f	61.62 ± 0.28d
VA14 × NS	10.18 ± 0.08d	1645.15 ± 1.58c	25.53 ± 0.37g	280.64 ± 1.25f	28.35 ± 0.17e	56.31 ± 0.41f
VA14 × MSS	15.89 ± 0.16b	2156.17 ± 2.62b	29.6 ± 0.29d	325.88 ± 1.27e	35.45 ± 0.16b	65.76 ± 0.62b
VA14 × SSS	21.46 ± 0.13a	3563.52 ± 2.57a	35.52 ± 0.26a	364.37 ± 2.22c	44.85 ± 0.20a	82.55 ± 0.82a
**Accession**						
VA3	9.14 ± 0.11c	221.62 ± 2.08c	30.44 ± 0.23a	131.49 ± 2.18c	33.81 ± 0.17b	59.27 ± 0.49b
VA12	8.58 ± 0.06b	1087.44 ± 2.49b	23.04 ± 0.28b	383.12 ± 1.67a	24.98 ± 0.26c	56.78 ± 0.27c
VA14	15.82 ± 0.05a	2454.67 ± 1.68a	30.22 ± 0.19a	323.63 ± 2.35b	36.22 ± 0.22a	68.21 ± 0.37a
**Salinity stress (SS)**						
NS	8.75 ± 0.15c	931.47 ± 3.01c	24.68 ± 0.17c	243.89 ± 1.37c	28.15 ± 0.23c	54.74 ± 0.28c
MSS	11.46 ± 0.16b	1140.84 ± 3.48b	27.57 ± 0.24b	280.55 ± 1.92b	31.15 ± 0.19b	60.31 ± 0.34b
SSS	14.27 ± 0.17a	1691.62 ± 2.68a	31.44 ± 0.18a	313.78 ± 1.83a	35.72 ± 0.17a	69.23 ± 0.28a
**Significance**						
Accession	***	***	***	***	***	***
SS	***	***	***	***	***	***
Accession × SS	***	***	***	***	***	***

SS, Salinity stress; NS, No saline water; MSS, Moderate salinity stress, SSS, Severe salinity stress, Values are means of six replicates and different letters are differed significantly by Duncan Multiple Range Test (***, P < 0.001).

**Fig 5:**
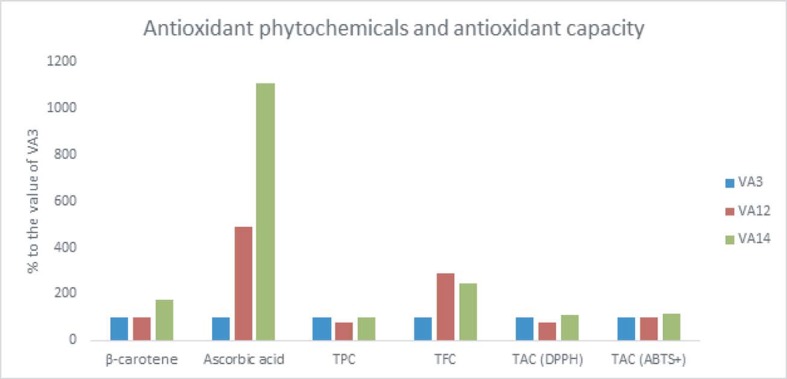
Response of antioxidant phytochemicals and antioxidant activities (% to the value of VA3) in three selected *A*. *tricolor* accessions; β-carotene (mg kg^-1^), Ascorbic acid (mg kg^-1^), TPC, total polyphenol content (GAE mg kg^-1^ dw); TFC, total flavonoid content (RE mg kg^-1^ dw); TAC (DPPH), total antioxidant capacity (DPPH) (TEAC mg kg^-1^ dw); TAC (ABTS^+^), total antioxidant capacity (ABTS^+^) (TEAC mg kg^-1^ dw).

Within accessions, TPC, β-carotene, TAC (DPPH), ascorbic acid, and TAC (ABTS^+^) was the highest in VA14 and VA12 had the highest TFC followed by VA14. Accession VA12 exhibited the lowest TAC (DPPH), β-carotene, TPC, and TAC (ABTS^+^). The VA14 had 74%, 10.07-fold, 46%, 7%, and 15% increase in TPC, β-carotene, TAC (DPPH), ascorbic acid, and TAC (ABTS^+^), respectively compared to VA3. Accession VA12 exhibited 3.9-fold and 190% increase in ascorbic acid and TFC, respectively, compared to VA3. (Fig 5).

**Fig 6:**
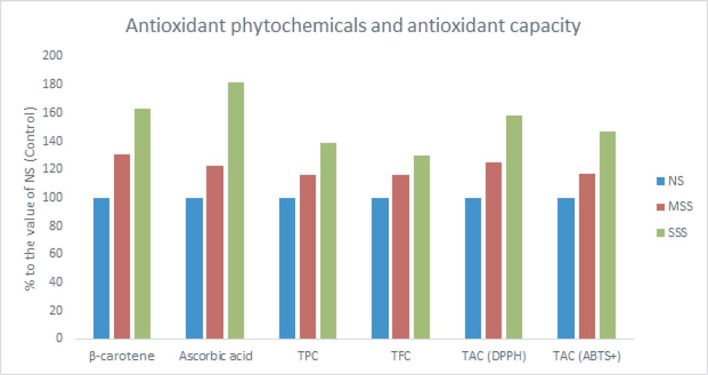
Response of antioxidant phytochemicals and antioxidant capacity (% to the value of NS or Control) under three salinity levels: NS or control (No saline water), MSS (Moderate salinity stress), SSS (Severe salinity stress) in three selected *A*. *tricolor* accessions; β-carotene (mg kg^-1^), Ascorbic acid (mg kg^-1^), TPC, total polyphenol content (GAE mg kg^-1^ dw); TFC. Total flavonoid content (RE mg kg^-1^ dw); TAC (DPPH), Total antioxidant capacity (DPPH) (TEAC mg kg^-1^ dw); TAC (ABTS^+^), Total antioxidant capacity (ABTS^+^) (TEAC mg kg^-1^ dw).

In our study, β-carotene, ascorbic acid, TPC, TFC, TAC (DPPH) and TAC (ABTS^+^) were significantly increased with the increment of salinity stress in the following order: NS < MSS < SSS. In MSS and SWS, β-carotene, ascorbic acid, TPC, TFC, TAC (DPPH) and TAC (ABTS^+^) were increased in (56%, 31%, 15%, 16%, 25% and 16%) and (112%, 115%, 39%, 30%, 58% and 47%) compared to NS, respectively (Fig 6). The highest β-carotene, ascorbic acid, TPC, TFC, TAC (DPPH) and TAC (ABTS^+^) were noticed in SSS while, the lowest β-carotene, ascorbic acid, TPC, TFC, TAC (DPPH) and TAC (ABTS^+^) were observed in NS.

Regarding the interaction of accession × salinity stress, VA14 under SSS exhibited the highest β-carotene, ascorbic acid, TPC, TAC (DPPH) and TAC (ABTS^+^), while VA12 under SSS had the highest TFC. In contrast, the lowest β-carotene, TPC, TAC (DPPH) and TAC (ABTS^+^) was observed in VA12 under NS, while VA3 under NS showed the lowest ascorbic acid and TFC. Only, ascorbic acid was significantly increased to the increment of salinity stress in all the accessions in the following order: NS < MSS < SSS. Higher β-carotene was observed in VA14 under MSS, VA12 under SSS, VA3 under SSS, VA14 under NS, while a high ascorbic acid was recorded in VA14 under MSS and VA14 under NS. Higher TPC was found in VA3 under SSS and VA3 under MSS, whereas, VA12 under MSS, VA14 under SSS and VA12 under NS had a high TFC. VA14 under MSS, VA3 under SSS, VA3 under MSS and VA3 under N showed a high TAC (DPPH), while VA14 under MSS, VA3 under SSS and VA12 under SSS had a high TAC (ABTS^+^).

### Correlation coefficients among antioxidant phytochemicals and antioxidant activity

Results of correlation studies are presented in Table 4. β-carotene showed highly significant interrelationships with ascorbic acid, TAC (DPPH), TAC (ABTS^+^) while, this trait had significant associations with TPC and TFC. Similarly, ascorbic acid revealed significant interrelationships with TPC, TFC, TAC (DPPH) and TAC (ABTS^+^). Both β-carotene and ascorbic acid played a vital role in the antioxidant activity of *A*. *tricolor*. TPC, TFC, TAC (DPPH) significantly interrelated among each other.

**Table 4:** Correlation coefficient for antioxidant phytochemical and antioxidant capacity in three selected *A*. *tricolor* accessions.

	β-carotene (mg kg^-1^)	Ascorbic acid (mg kg^-1^)	Total polyphenol content (GAE mg kg^-1^ dw)	Total flavonoid content (RE mg kg^-1^ dw)	Total antioxidant capacity (DPPH) (TEAC mg kg^-1^ dw)	Total antioxidant capacity ABTS+) (TEAC mg kg^-1^ dw)
β-carotene		0.94**	0.68*	0.71*	0.82**	0.97**
AsA			0.66*	0.72*	0.76*	0.75*
TPC				0. 77*	0.95**	0.85**
TFC					0.84**	0.83**
TAC (DPPH)						0.96**

AsA, Ascorbic acid; TPC, Total polyphenol content; TFC, Total flavonoid content; TAC (DPPH), Total antioxidant capacity (DPPH); TAC (ABTS^+^), Total antioxidant capacity (ABTS^+^)

*significant at 5% level

** significant at 1% level, (n = 6)

## Discussion

Amaranth was considered as the inexpensive leafy vegetables and its cultivation was also limited to Africa, South-East Asia and South America. Recently, amaranth spread over worldwide and its production and consumption have been remarkably increased due to the presence of excellent natural antioxidants such as minerals, antioxidant leaf pigments, carotenoids, vitamins, phenolics and flavonoids. These natural antioxidants have proven health benefits as they detoxify ROS in the human body and involve in defense against several diseases such as cancer, atherosclerosis, arthritis, cataracts, emphysema, retinopathy, neuro-degenerative and cardiovascular diseases [14–17]. Amaranthus species have higher mineral concentrations than commonly consumed leafy vegetables, such as spinach, lettuce and kale [36]. In *A*. *tricolor*, iron and zinc content is higher than that of the leaves of cassava [37] and beach pea [38]. The U.S. Department of Agriculture’s National Nutrient Database for Standard Reference [39] lists a serving size of spinach as 30 g fresh weight FW (1 cup). As *Amaranthus* has higher mineral concentrations than spinach so, a serving size of leaves of 30 g FW is enough for nutritional sufficiency. In general, leafy vegetables are susceptible salt stress but amaranth is salt tolerant plant [10]. This study comprehensively evaluates the effects of varying levels of salinity stress on contents of nutrients, minerals, dietary fiber, phytochemicals and antioxidant activities of *A*. *tricolor* accessions. Our results for the first time demonstrated that soil salinity stress up to certain level significantly augment almost all these biochemical parameters in leaves of *A*. *tricolor*. However, the responses of these parameters to salinity varied among the accessions of *A*. *tricolor*. Altered proteomes, enhanced vitamins and glycine betaine contents in salinity stressed Amaranthus have previously been reported [10, 40, 41].

One of the interesting findings of our study is that salinity stresses 50 mM and 100 mM NaCl concentrations significantly improved protein, ash, energy, dietary fiber, Ca, Mg, Fe, Mn, Cu, Zn, Na, β-carotene, ascorbic acid, total polyphenol content (TPC), total flavonoid content (TFC), total antioxidant capacity (TAC) (DPPH) and total antioxidant capacity (TAC) (ABTS^+^) (Tables 1, 2 and 3) in leaves of *A*. *tricolor* compared to control condition. control. Salt-stressed *A*. *tricolor* leaves also showed remarkable increment in protein, ash, energy, dietary fiber, minerals and functional antioxidant phytochemicals compared to normal cultural condition (Figs 2, 4 and 6). To the best of our knowledge, this is the first report of remarkable and progressive improvement of the proximate, nutritional and functional antioxidant phytochemicals contents in *A*. *tricolor* under salinity stresses compared to non-saline soil conditions.

Another interesting finding of this study is that responses of biochemical contents in different *A*. *tricolor* accessions were different. The accession, VA14 performed better in terms of protein, ash content, and energy content, respectively compared to the accession VA3. Similarly, the accession VA12 performed better in relation to carbohydrates and energy, respectively than the accession VA3 (Table 1). The maturity could have a great impact on the moisture content of *A*. *tricolor* leaves. The moisture contents obtained in this investigation were in full agreement with the reports on sweet potato leaves by Sun et al. [42]. Fats are sources of omega-3 and omega-6 fatty acids. It helps in the digestion, absorption, and transport of fat-soluble vitamins A, D, E, and K. Sun et al. [42] observed similar results from sweet potato leaves where they mentioned that fat involved in the insulation of body organs and in the maintenance of body temperature and cell function.

As lower moisture contents of leaves are associated with higher dry matter, the salt-stressed plant yielded higher dry matter compared to control or NS. The highest contents of protein, ash, energy and dietary fiber at SSS conditions and the lowest values of these plant parameters in the control or NS appears that protein, ash, energy and dietary fiber contents in *A*. *tricolor* increased by salinity stress in a dose-dependent manner. The increment of protein, ash, energy and dietary fiber contents in *A*. *tricolor* at MSS and SSS could be contributed to human diet in the communities of saline prone area compared to non-saline area. Dietary fiber has a significant role in palatability, digestibility and remedy of constipation [23]. Vegetarian and poor people in many least developed Asian and African countries used *A*. *tricolor* as a source of protein. Plants cultivated in SSS had progressively higher energy than those of MSS and control or NS. However, these differences may not impact significantly on energy contribution to the human body as low amounts of this vegetable consumed in a day. Like other leafy vegetables, the low carbohydrate content of *A*. *tricolor* may not have a significant impact on carbohydrate contribution to the human body considering the low amount of vegetable uptake per day and a very high daily requirement for the human body.

A remarkable observation of this investigation is that the content of protein is increased with plants grown in higher doses of salinity. However, the trend of fat contents in plants under salinity treatment was just opposite to the contents of protein. It indicates that both salinity and accession had a complex influence on carbohydrate contents in *A*. *tricolor* plants. In an earlier study, Petropoulos et al. [11] demonstrated ameliorate response in carbohydrates, protein and fat content in *Cichorium spinosum* under salinity stress. Salt stress increased the protein, dietary fiber, energy, ash and carbohydrates content and decreased moisture and fat content of *A*. *tricolor* accessions. Therefore, amaranth produced saline prone area and coastal belt could contribute as a good source of protein and fiber in the human diet.

We observed that salinity stress influences the mineral compositions of *A*. *tricolor* accessions. Among the tested accessions, VA14 could be consider as Ca, K, Fe, Mn, Cu and Zn enrich accession, VA3 as Mg and VA12 as Na enrich accessions (Table 2). In *A*. *tricolor*, iron and zinc content is higher than that of the leaves of cassava [37] and beach pea [38]. Similarly, Jimenez-Aguiar and Grusak [36] reported high Fe, Mn, Cu and Zn (fresh weight basis) in different *A*. *spp*. including *A*. *tricolor*. They also reported that Amaranths had higher Zn content than black nightshade, spinach and kale; more Fe and Cu content than kale. Across salinity stress, Ca, Mg, Fe, Mn, Cu, Zn and Na content were sharply and significantly increased with the increment of salinity stress in the following order: NS or control < MSS < SSS. In contrast, it was noted that the severity of salinity stress leads to a significant reduction in K content in the following order: NS or control > MSS > SSS (Table 2). These results were fully agreed with the findings of Petropoulos et al. [11] that observed similar increment in Ca, Mg, Fe, Mn, Zn and Na and decrement in K content in *C*. *spinosum* leaves. They mentioned the high content of Na should be attributed to fertilizer application and salinity treatments and suggested that the species uses Na accumulation as a means to alleviate adverse effects of salinity. With the severity of salinity stress, all the macro and micro elements except K showed increasing trend, while K showed the declining trend with the severity salinity stress. For this, amaranth cultivated in salinity prone area and coastal belt could contribute as a good source of minerals in human diet compared to normal cultivation practices.

An important finding of the current study is that β-carotene, ascorbic acid, total polyphenol content (TPC), total flavonoid content (TFC) and total antioxidant capacity (TAC) of *A*. *tricolor* leaves were significantly augmented by the salt stress at certain level (Table 3). These important phytochemicals content were remarkably influenced by the accessions and accession × salt concentration interactions. The accessions VA14 could be consider as TPC, β-carotene, TAC, ascorbic acid, antioxidant enrich accession and VA12 as flavonoid enrich accession. In the present study, we found great variations in the tested accessions in terms of TPC, β-carotene, TFC, TAC (DPPH) and TAC (ABTS^+^) in different salinity levels (Table 3). Similarly, Alam et al. [12] reported pronounced variations in TFC, TPC, and TAC in different purslane accessions.

In our study, β-carotene, ascorbic acid, TPC, TFC, TAC (DPPH) and TAC (ABTS^+^) were significantly increased with the increment of salinity stress in the following order: NS < MSS < SSS. VA14 under SSS exhibited the highest β-carotene, ascorbic acid, TPC, TAC (DPPH) and TAC (ABTS^+^), while VA12 under SSS had the highest TFC. In contrast, the lowest β-carotene, TPC, TAC (DPPH) and TAC (ABTS^+^) was observed in VA12 under NS, while VA3 under NS showed the lowest ascorbic acid and TFC. When plants fall under salinity stress, reactive oxygen species (ROS) are produced as a results of oxidative stress. ROS induces harmful effects on plant cells. As a result, defenses against ROS are activated by generation of an array of nonenzymatic antioxidants such as ascorbic acid (AsA) and β-carotene [43]. Salinity stress induces mevalonic acid pathway which are responsible for biosynthesis of abscisic acid (ABA) from carotenoids to counteract the osmotic stress and regulate normal plant growth and development [44]. Therefore, salinity stress enhances the accumulation of β-carotene due to induction of ABA. AsA and αtocopherols play a crucial role in quenching intermediate/excited reactive forms of oxygen molecule directly or through catalysis of enzymes. AsA scavenges ROS (OH, SOR and ^1^O_2_ directly and reduces H_2_O_2_ to water through ascorbate peroxidase reaction [45]. Antioxidant ascorbate and total carotenoid had vital role in counterbalancing oxidative stress and manipulating homeostasis of ROS in plants [46]. Wouyou et al. [41] observed ameliorate response of vitamin A and vitamin C at 90 mM NaCl concentration in *Amarantus cruentus* leaves. Similarly, Petropoulos et al. [11] found an elevated response to phenolics, flavonoids and antioxidant activity with the increase in salt stress in *Cichorium spinosum*. Alam et al. [12] observed that in purslane, different doses of salt concentrations increased total polyphenol content (TPC); total flavonoid content (TFC) and FRAP activity by 8–35%, 35% and 18–35%, respectively. Lim et al. [13] reported that buckwheat treated with 10, 50, and 100 mM after 7 d of cultivation had 57%, 121% and 153%, respectively, higher phenolic content than that of the control. Ahmed et al. [47] reported the increment of phenolics and TAC (FRAP) with increasing NaCl concentrations in barley. In contrast, Neffati et al. [48] found decrement in polyphenols and TAC (DPPH) with increasing NaCl concentrations in coriander.

The increment of TPC, TFC and TAC of *A*. *tricolor* in response to salinity stress may be due to increase in major phenolic compounds like salisylic acid, gallic acid, vanilic acid, *p*-hydroxybenzoic acid, chlorogenic acid, *m*-coumaric acid, *trans-*cinnamic acid, iso-quercetin and rutin [35]. Previous studies have shown that biotic and abiotic stress stimulated phenylpropanoid pathway which accelerated the generation of most phenolic compounds [49, 50]. Stress-plants induce endogenous plant hormones like jasmonic acid and its methylated derivate (methyl jasmonic acid) [51]. These hormones sequentially induce phenylpropanoid pathway enzymes, including phenylalanine ammonia lyase (PAL) [52]. These enzymes accumulated the phenolic compounds.

The β-carotene showed highly significant interrelationships with ascorbic acid, TAC (DPPH), TAC (ABTS^+^) while, this trait had significant associations with TPC and TFC. Similarly, ascorbic acid revealed significant interrelationships with TPC, TFC, TAC (DPPH) and TAC (ABTS^+^) (Table 4). ascorbic acid played a vital role in the antioxidant activity of *A*. *tricolor*. TPC, TFC, TAC (DPPH) significantly interrelated among each other. Polyphenols and flavonoids of *A*. *tricolor* leaf establishing strong antioxidant activity. Alam et al. [12] reported the significant correlation of carotenoids, TPC, TFC with TAC (FRAP) in salt-stressed purslane.

In conclusion, a significant increment in protein, ash, energy, dietary fiber, carbohydrates, Ca, Mg, Fe, Mn, Cu, Zn, Na, β-carotene, ascorbic acid, TPC, TFC, TAC (DPPH) and TAC (ABTS^+^) in *A*. *tricolor* leaves were observed under salinity stress. All the nutritional values of *A*. *tricolor* leaves under MSS and SSS remarkably high compared to corresponding control or NS values which could be a valuable food source in modern diets and contribute considerably to human health. Furthermore, salt-stress also enhanced the contents of protein, ash, energy, dietary fiber, Ca, Mg, Fe, Mn, Cu, Zn, Na, β-carotene, ascorbic acid, TPC, TFC in leafy vegetables *A*. *tricolor*. The vitamins, phenolics and flavonoids showed a good antioxidant activity due to positive and significant interrelationships with TAC. Our results suggest that *A*. *tricolor* cultivated under salinity stress could be contributed to a high nutritional quality of the final product in terms of nutrients, minerals, vitamins and antioxidant profiles. Therefore, *A*. *tricolor* could be considered as a promising alternative crop for farmers, especially in salinity-prone areas and the coastal belts in tropical and sub-tropical countries.
